# Measurement of Systolic Blood Pressure Using POCUS With Color Doppler Compared to with an Intraarterial Line

**DOI:** 10.24908/pocusj.v10i02.19281

**Published:** 2025-11-17

**Authors:** Henry Mayo-Malasky, Daniel Ying, Alekhya Bukkuri, Paul H. Mayo

**Affiliations:** 1 Division of Pulmonary, Critical Care, and Sleep Medicine, LIJ/NSUH Northwell, Donald and Barbara Zucker School of Medicine at Hofstra/Northwell, Hempstead, NY USA; 2 Division of Pulmonary, Critical Care, and Sleep Medicine Winthrop Hospital New York University School of Medicine, Winthrop Division Mineola, NY USA

**Keywords:** POCUS, Point of care ultrasound, Blood pressure, Color Doppler, Comparison of methods

## Abstract

**Background:**

In some clinical circumstances, it may be difficult to accurately measure systolic blood pressure (SBP) using direct auscultation technique or an automated oscillometric cuff pressure device. As an alternative method, this study compared the measurement of SBP using point of care ultrasound (POCUS) with color Doppler to the measurement of SBP using an intraarterial catheter.

**Methods:**

Study subjects were 50 patients in an intensive care unit who had an intraarterial catheter placed for monitoring blood pressure. The intraarterial catheter systolic pressure was recorded and compared to the contemporaneous measurement of SBP using POCUS with color power Doppler (CPD). The operator placed the Doppler sample volume over the brachial artery with ipsilateral inflation of a prepositioned upper arm blood pressure cuff that was inflated sufficiently to ablate blood flow in the target artery. The blood pressure cuff was then deflated until there was return of CPD signal in the brachial artery. At this moment, the corresponding blood pressure was noted on a sphygmomanometer attached to the blood pressure cuff. The values of the two methods were compared using standard statistical technique.

**Results:**

The intraarterial systolic pressures and CPD systolic pressures by POCUS were well correlated with a Pearsons correlation coefficient of 0.96. Bland-Altman analysis of bias and limits of agreement indicated that the POCUS with CPD measurement was sufficiently accurate to have clinical utility.

**Conclusions:**

The use of POCUS with CPD to measure SBP may have utility in situations where direct auscultation or automated oscillometeric cuff pressure measurements may be unreliable.

## Introduction

Adequate blood pressure is a key outcome variable of successful resuscitation. In some circumstances, accurately measuring systolic blood pressure (SBP) in a patient who is critically ill can be challenging. This difficulty may arise during rapid response situations due to high ambient noise environments interfering with auscultation, patient-related factors affecting non-invasive blood pressure determination, or the initial absence of intraarterial blood pressure monitoring [1,2]. Here we report on the correlation of SBP determined using point of care ultrasound (POCUS) with color power Doppler (CPD) compared to SBP measured with an indwelling intraarterial catheter. This method may have utility in circumstances when other methods of measurement may be inaccurate pending insertion of an intraarterial catheter.

## Methods

This study was conducted at the Long Island Jewish Hospital, which is a 580-bed teaching hospital with a 14-bed medical intensive care unit (MICU) staffed by pulmonary/critical care medicine fellows and fulltime faculty attendings of the Division of Pulmonary, Critical Care, and Sleep Medicine. This study was approved by the Feinstein Institute for Medical Research Institutional Review Board which waived the requirement for informed consent (IRB#19-0440).

### Study Design

The study subjects were a non-sequential convenience sample that were selected based upon the availability of one of the investigators and two independent observers who were residents or fellows on MICU rotation. Any patient with an intraarterial catheter inserted by the clinical care team for blood pressure monitoring was eligible for inclusion. Exclusion criteria included factors that prevented use of a blood pressure cuff on the arm contralateral to the intraarterial catheter, such as the presence of an arteriovenous conduit used for hemodialysis, history of axillary lymph node dissection, wounds or dressings on the upper arm, absence of the arm, orthopedic or cutaneous injury to the arm, and any visual evidence of respirophasic variation of systolic pressure of the arterial waveform displayed on the bedside MICU monitor.

The accuracy of the intraarterial blood pressure monitoring system was verified by correctly zeroing the system, subjecting it to a flush test to exclude dampening of the signal, and using a standard calibrated pressure transducer (Transpac, ICU Medical, San Clemente, CA). An appropriately sized blood pressure cuff was applied to the arm contralateral to the intraarterial catheter with the cuff indicator placed directly over the brachial artery in the bicipital groove just above the antecubital fossa. The sphygmomanometer, of standard spring-loaded design (Welch Allyn 767-Series Wall Sphygmomanometer, Welch Allyn, Skaneateles Falls, NY) was used to measure the cuff blood pressure. The sphygmomanometer was factory calibrated with a lifetime guarantee of accuracy.

Using a 7.5 MHz linear vascular ultrasonography probe (Venue, GE HealthCare, Chicago, IL), one investigator acquired an image of the brachial artery and positioned a CPD sample volume over the vessel. Most studies were performed using a CPD scale of 1.00 kHz (the machine preset); however, the operator had the option of reducing the CPD scale to 0.50 kHz to optimize the color signal. The investigator then inflated the blood cuff until there was an absence of blood flow in the target artery indicated by lack of CPD signal in the vessel. The cuff pressure was then reduced by approximately 1 mm Hg per second until blood flow was detected within the brachial artery with CPD.

Two independent observers who had been briefed on their responsibilities simultaneously watched the sphygmomanometer gauge. When the investigator announced visual detection of color Doppler signal—which indicated return of brachial artery blood flow during blood pressure cuff deflation—the two observers noted the corresponding blood pressure on the sphygmomanometer gauge. They were not permitted to verbally report the value but were required to write it while blinded to each other's value. The arterial pressure at the time of their report was noted by the investigator. All data was then entered into a deidentified form in a REDcap (Redcap, Nashville, TN) database for subsequent analysis. Basic demographic and disease related data were collected for each patient.

### Statistical Analysis

The correlation between the intraarterial SBP and POCUS with CPD SBP was quantified by calculating Pearson's correlation coefficient. A Bland–Altman analysis was used to evaluate the level of agreement between the intraarterial SBP and POCUS with CPD SBP. The interobserver variability of the sphygmomanometer SBP was quantified by calculating Pearson's correlation coefficient comparing the two values. Statistical analysis was performed using SigmaStat software.

## Results

**Figure 1.**
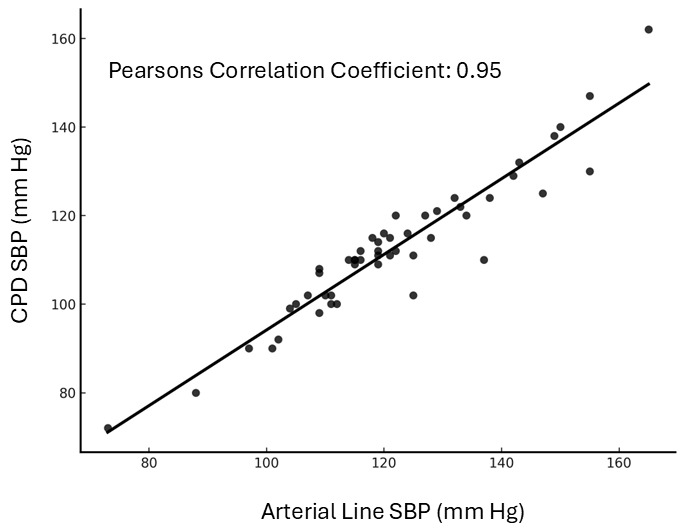
Correlation of systolic blood pressure (SBP) measured with intraarterial line to SBP measured with color power Doppler (CPD) by point of care ultrasound (POCUS).

**Figure 2.**
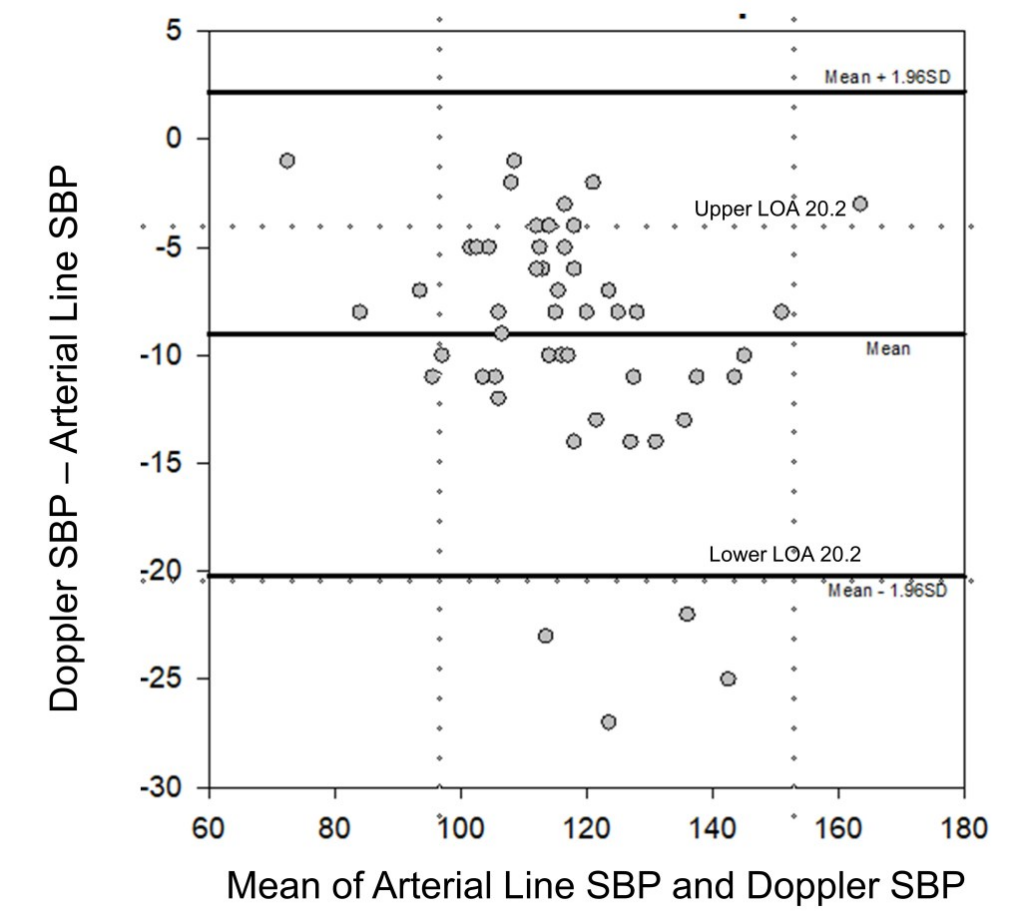
Bland-Altman plot of systolic blood pressure (SBP) measured with the intraarterial line and SBP measured with color power Doppler (CPD) by point of care ultrasound (POCUS).

**Figure 3.**
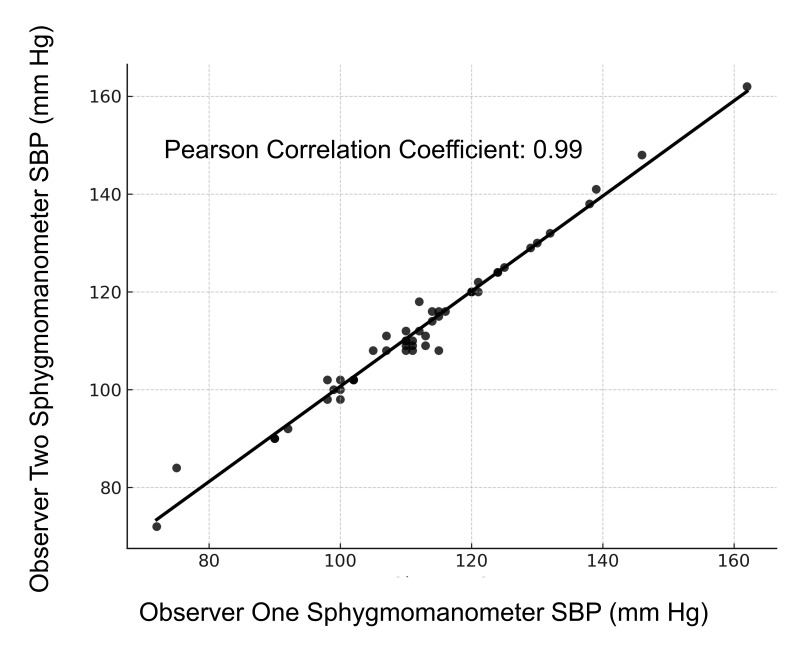
Interobserver variability of the observed systolic blood pressure (SBP) from the sphygmomanometer-detected SBP.

A total of 50 subjects were enrolled in the study. Demographics, disease characteristics, and vasopressor dose at the time of the measurements are presented in Table 1. The intraarterial SBP and the CPD SBP measurements are compared in Figure 1 with Pearson's coefficient correlation of 0.95. A Bland-Altman analysis of the data is presented in Figure 2 with mean difference (bias) of -9.0 mm Hg and standard deviation of differences of 5.7 mm Hg. Four pairs of values were outside of the limits of agreement; in both, the arterial pressure was higher than the POCUS with CPD measurement. The interobserver variability of the sphygmomanometer measurements between the two blinded observers are presented in Figure 3 with a Pearson's coefficient correlation of 0.99—indicating that interobserver variability of the sphygmomanometer value was minimal.

**Table 1.** Patient characteristics (n = 50).

Patient demographics	Value
Patient age (years, S.D.)	62.2, 20.1
Sex (male/female)	28/22
Site of intraarterial line (%)	Axillary 33 (66)
	Radial 14 (28)
	Femoral 2 (4)
	Brachial 1 (2)
**Primary disease requiring intensive**	
Hemodynamic failure	27 (54)
Respiratory Failure	11 (22)
Neurological/metabolic disarray	12 (24)
**Vasopressor use agent/dose**	
No vasopressors (%)	23 (46)
Norepinephrine (%)	17 (34)
Dose (ug/kg/min) (mean, range)	0.40, 0.03–0.7
Vasopressin (%)	9 (18)
Dose (units/min)	0.04 uniform dose
Phenylephrine (%)	1 (2)
Dose (ug/kg/min)	0.5

## Discussion

The results of this study indicate that the SBP detected by POCUS using CPD is well correlated with that measured with an intraarterial catheter. The bias is acceptable for clinical applications with 92% of values within the limits of agreement, so the two methods appear to be comparable. Four data points of 50 are outside the limits of agreement, indicating good consistency between the two methods. The accuracy of the POCUS with CPD measurement was unaffected by the use of vasopressor agents or the position of the intraarterial line. Interobserver variability of the sphygmomanometer measurement was minimal.

Adequate blood pressure is an important end point for resuscitation from hemodynamic failure. In most circumstances, it is measured with automated cuff systems before admission to the intensive care unit; or, when indicated, via an intraarterial catheter following admission to the intensive care unit. We propose that the POCUS with CPD method may be useful in specific circumstances such as upon return of spontaneous circulation following cardiac arrest or during a rapid response situation involving a patient with severe hemodynamic failure. During these hectic and often noisy events, there may be difficulty in obtaining accurate blood pressure by auscultation; the value obtained with an automated cuff may be of questionable value, and intraarterial pressure monitoring may not be readily available. As adequate systolic pressure is a key goal of the care team during initial treatment, the POCUS with CPD method offers a simple and reliable means of detecting SBP during a challenging care period.

The investigators used POCUS with CPD as the Doppler method due to its ease of use and availability on the full-service machine that was used in the MICU. An alternative approach is to use standard color Doppler with a reduction of the color Doppler scale to optimize the detection of blood flow in the brachial artery. New generation ultraportable ultrasound machines may not have CPD capability, so the operator may need to downscale the color Doppler to measure SBP. Anecdotally, we have used this approach with similar results to using CPD. The low cost and ease of use of the new ultraportable devices combined with increasing widespread acceptance of POCUS by the emergency medicine, hospitalist, and critical care communities, will allow color Doppler measurement of SBP in specific clinical situations. Standard color Doppler is angle dependent, whereas CPD is not. The operator who uses standard color Doppler should be cognizant of its angle dependency and optimize the insonation angle to improve signal quality. This constitutes another useful application for ultraportable devices in addition to the full-service machines used in the MICU and emergency department.

Another potential application of the POCUS with CPD technique might be in the MICU to verify that the automated blood pressure cuff is measuring accurate SBP. In this case, the method might offer a non-invasive method to serve as a “gold standard” to which the suspect automated blood pressure method may be compared.

Using POCUS with color Doppler to detect SBP shares similarities with dedicated handheld Doppler devices that are used to detect arterial blood flow [3]. In both cases, the operator listens for an audible signal of blood flow derived from the Doppler signal while deflating the blood pressure cuff to identify the presence of blood flow. We did not compare the two methods as the POCUS color Doppler method was more practical and widely available.

This study has several limitations. The CPD measurement was made from the arm contralateral to the intraarterial measurement. Asymmetric brachial artery pressures are associated with a variety of conditions such as aortic dissection, extensive asymmetric atherosclerosis, and with arterial compression or anomalous anatomic variants. To our knowledge, none of these were present in our study population. While the sphygmomanometer reading was reported by two separate observers who were blinded to one another, only one observer reported on the CPD signal. The study population did not include patients at the extremes of low or high SBP (the lowest was 73 mm Hg, and the highest was 165 mm Hg). The size of the blood pressure cuff relative to the circumference of the subject's arm was determined by “eyeball” assessment. Ideally, the cuff size would have been standardized. We did not compare the measurement of SBP using auscultation or using an automated oscillometric device to SBP measured with CPD. This will require further investigation. Four measurements of SBP using POCUS with CPD were well below that measured with the intraarterial line. We have no clear explanation for this. The other 46 measurement pairs were sufficiently well correlated as to have clinical utility [4]. This study did not include patients who were peri-morbid or in the immediate period following return of spontaneous circulation after cardiac arrest. The POCUS with CPD SBP technique would have special application in this population. Whether our results would apply to these clinical scenarios will require further study.

## Conclusions

SBP may be determined with acceptable accuracy by observing the return of CPD signal by POCUS in the brachial artery when deflating a blood pressure cuff. This technique may have specific application in pressured clinical environments including during post cardiac arrest and in rapid response team scenarios. As such, it represents another application of utility to frontline POCUS clinicians.

## References

[1] Tatliparmak AC, Yilmaz S. Agreement of Oscillometric and Auscultatory blood pressure measurement methods: An ambulance noise simulation study. The American Journal of Emergency Medicine. 2023;67:120–5. doi: 10.1016/j.ajem.2023.02.022. 36870252

[2] Picone DS, Schultz MG, Otahal P, Aakhus S, Al-Jumaily AM, Black JA, Bos WJ, Chambers JB, Chen CH, Cheng HM, Cremer A. Accuracy of cuff-measured blood pressure: systematic reviews and meta-analyses. Journal of the American College of Cardiology. 2017;70(5):572–86. doi: 10.1016/j.jacc.2017.05.064 28750701

[3] Vehrs, P.R., Richards, S., Blazzard, C., Hart, H., Kasper, N., Lacey, R., Lopez, D. and Baker, L. Use of a handheld Doppler to measure brachial and femoral artery occlusion pressure. Frontiers in Physiology, 2023;14: p.1239582. doi: 10.3389/fphys.2023.1239582 37664423 PMC10470651

[4] O'Brien E, Pickering T, Asmar R, Myers M, Parati G, Staessen J, Mengden T, Imai Y, Waeber B, Palatini P, Gerin W. Working Group on Blood Pressure Monitoring of the European Society of Hypertension International Protocol for validation of blood pressure measuring devices in adults. Blood Pressure Monitoring. 2002;7(1):3–17. doi: 10.1097/00126097-200202000-00002 12040236

